# The RS4;11 cell line as a model for leukaemia with t(4;11)(q21;q23): Revised characterisation of cytogenetic features

**DOI:** 10.1002/cnr2.1207

**Published:** 2019-08-07

**Authors:** Denise Ragusa, Evgeny M. Makarov, Oliver Britten, Daniela Moralli, Catherine M. Green, Sabrina Tosi

**Affiliations:** ^1^ Division of Biosciences, College of Health and Life Sciences, Institute of Environment, Health and Societies Brunel University London Uxbridge UK; ^2^ Genome Engineering and Maintenance Network, Institute of Environment, Health and Societies Brunel University London Uxbridge UK; ^3^ Chromosome Dynamics, Wellcome Centre for Human Genetics University of Oxford Oxford UK

**Keywords:** clonal evolution, KMT2A, leukaemia, MLL, RS4;11, t(4;11)(q23;q21)

## Abstract

**Background:**

Haematological malignancies harbouring rearrangements of the *KMT2A* gene represent a unique subtype of leukaemia, with biphenotypic clinical manifestations, a rapid and aggressive onset, and a generally poor prognosis. Chromosomal translocations involving *KMT2A* often cause the formation of oncogenic fusion genes, such as the most common translocation t(4;11)(q21;q23) producing the KMT2A‐AFF1 chimera.

**Aim:**

The aim of this study was to confirm and review the cytogenetic and molecular features of the *KMT2A*‐rearranged RS4;11 cell line and put those in context with other reports of cell lines also harbouring a t(4;11) rearrangement.

**Methods and Results:**

The main chromosomal rearrangements t(4;11)(q21;q23) and i(7q), described when the cell line was first established, were confirmed by fluorescence in situ hybridisation (FISH) and 24‐colour karyotyping by M‐FISH. Additional cytogenetic abnormalities were investigated by further FISH experiments, including the presence of trisomy 18 as a clonal abnormality and the discovery of one chromosome 8 being an i(8q), which indicates a duplication of the oncogene *MYC*. A homozygous deletion of 9p21 containing the tumour‐suppressor genes *CDKN2A* and *CDKN2B* was also revealed by FISH. The production of the fusion transcript *KMT2A‐AFF1* arising from the der(11)t(4;11) was confirmed by RT‐PCR, but sequencing of the amplified fragment revealed the presence of multiple isoforms. Two transcript variants, resulting from alternative splicing, were identified differing in one glutamine residue in the translated protein.

**Conclusion:**

As karyotype evolution is a common issue in cell lines, we highlight the need to monitor cell lines in order to re‐confirm their characteristics over time. We also reviewed the literature to provide a comparison of key features of several cell lines harbouring a t(4;11). This would guide scientists in selecting the most suitable research model for this particular type of *KMT2A*‐leukaemia.

## 1 INTRODUCTION

Leukaemia harbouring rearrangements of the *KMT2A* gene (formerly known as *MLL*/*mixed‐lineage leukaemia* and also known as *HRX* or *TRX1*) represent a unique subtype of acute leukaemia, characterised by a rapid and aggressive onset with generally poor prognosis. *KMT2A* rearrangements can give rise to different cellular phenotypes, with affected cells showing an interesting lineage heterogeneity, hence the designation of “mixed‐lineage”. Rearrangements involving *KMT2A* are often found in de novo and DNA topoisomerase II inhibitor therapy‐related myeloid and lymphoblastic acute leukaemias, with varying incidence according to type and age.^1^ Overall, *KMT2A* rearrangements account for 10% of all acute leukaemias^2^ but are predominantly found in infants between the age of 0 and 2 diagnosed with acute lymphoblastic leukaemia (ALL; 70%‐80% of cases^3^, ^4^) and in therapy‐related acute myeloid leukaemia (AML) patients (up to 70% of cases).^5^

Chromosomal translocations are common rearrangements in *KMT2A*‐leukaemia,^6^ whereby the exchange of genetic material between chromosomes brings the N‐terminus of *KMT2A* to fuse in‐frame with the C‐terminus of a partner gene.^7^, ^8^ The breakpoint region of *KMT2A* covers approximately 8 kbp between exons 7 and 11.^9^ More than 90 partner genes for *KMT2A* have been identified, forming the so‐called “*MLL* recombinome.” The most common translocation partners are *AFF1* (previously known as *AF4*) on 4q21, *MLLT3* (*AF9*) on 9q22, *ELL* on 19p13.1, *MLLT1* (*ENL*) on 19p13.3, *MLLT10* (*AF10*) on 10p12, and *MLLT4* (*AF6*) on 6q27, giving rise to t(4;11)(q21;q23), t(9;11)(q22;q23), t(11;19)(q23;p13.1), t(11;19)(q23;p13.3), t(10;11)(p12;q23), and t(6;11)(q27;q23), respectively.^1^ The in‐frame fusion of *KMT2A* and a partner gene produces chimeric proteins with oncogenic activity, which largely depends on the retained domains of *KMT2A* and the characteristics of the fusion partner.^10^

The most common translocation in the *MLL* recombinome is the t(4;11)(q21;q23), which produces the KMT2A‐AFF1 chimeric protein.^1^, ^11^ The phenotype is mainly B‐ALL, with rare cases of AML. These leukaemic cells possess a biphenotypic profile, as they co‐express lymphoid and myeloid markers while maintaining a lymphoblastic morphology, suggesting that the original malignant clone arises from an early lymphoid/myeloid precursor.^12^

Leukaemia initiation by *KMT2A* rearrangements is thought to occur via an improper expression and regulation of Hox genes.^13^ The KMT2A protein is a homologue of the trithorax protein in *Drosophila melanogaster* 14 and functions as a transcriptional activator and regulator of Hox genes during embryogenesis and haematopoiesis.^15^, ^16^ AFF1 is a nuclear protein acting as transcriptional regulator involved in haematopoietic development of lymphoid precursors.^17^ The KMT2A‐AFF1 fusions are capable of initiating and maintaining an erroneous programme of transcription with oncogenic consequences.^18^

It is a topic of debate whether KMT2A fusions alone are sufficiently powerful to cause the disease, contradicting the “multi‐hit model” that applies to most leukaemias.^19^ Supporting the prenatal origin of leukaemia, alterations of the *KMT2A* gene have been documented in utero.^20^ One of the most remarkable features of infant *KMT2A*‐leukaemia is the extraordinarily short latency and the limited number of secondary somatic mutations,^21^ indicating that only a small number of additional events may be necessary to initiate the malignancy, if at all.^22^, ^23^ Mutations in *FLT3*, *KRAS*, and *NRAS* have been proposed as “second hits” drivers for leukaemogenesis of *KMT2A*‐leukaemias.^24^, ^25^

A reliable in vivo model faithfully mimicking the disease is still lacking (reviewed in Ottersbach et al^26^). Interestingly, murine models so far have achieved a transient production of KMT2A‐AFF1 proteins but failed to express the phenotype observed in humans.^27^, ^28^, ^29^ Only recently, the development of such models is becoming increasingly closer to clinical phenotypes.^30^, ^31^ Therefore, in vitro models have been at the forefront of research into *KMT2A*‐leukaemia. Although at least 16 cell lines with the t(4;11) have been described, only four have been appropriately authenticated.^32^, ^33^ In this article, we focus on the re‐visitation of the RS4;11 cell line, which was first established by Stong et al^34^ from a 32‐year‐old female patient with relapsed ALL. The initial karyotype described a t(4;11)(q21;q23) and the presence of an i(7q).

Our work focuses on the characterisation of cytogenetic and molecular aspects of RS4;11 in comparison with previous work from other groups. As karyotype evolution is common in extended cell cultures,^32^ we highlight the need for a constant monitoring and validation of cell lines to be used as a reliable research model. We also provide a brief summary of characteristics of other *KMT2A*‐*AFF1*‐positive cell lines and comment on their suitability to advance our understanding of *KMT2A*‐driven leukaemogenesis.

## 2 MATERIALS AND METHODS

### 2.1 Cell lines

The cell lines RS4;11 (ATCC CRL‐1873™) and Farage (ATCC CRL‐2630™) were grown in RPMI 1640 (Gibco, Paisley, UK) supplemented with 10% foetal bovine serum (FBS) (Gibco) and 1% penicillin/streptomycin (100 UmL^−1^/μgmL^−1^) (Gibco), and incubated at 37°C in 5% CO_2_. Cells were passaged every 48 hours.

### 2.2 Fluorescence in situ hybridisation (FISH) and M‐FISH

Metaphase chromosomes were obtained by adding colcemid (10 μg/mL; Gibco, Paisley, UK) to cell cultures 1 hour before harvesting according to well‐established methods.^35^ The commercially available DNA probes XCAP 7 long, XCAP 7 short, XL 7q22/7q36, XL MLL Plus, XL CDKN2A (Metasystems, Altlussheim, Germany), WCP18, WCP8, WCPX (Cambio, Cambridge, UK), and a BAC‐derived DNA probe RP11‐195E4 (BACPAC Resources Center, Oakland, US) labelled in house were used for fluorescence in situ hybridisation (FISH) experiments. The RP11‐195E4 spans the region 8q24.3 (141,431,223 to 141,608,396). Detailed information on all probes is reported in Table 1. FISH was performed according to the manufacturer's instructions (Metasystems) with minor modifications or according to existing protocols^35^; 8 μL of probe mixture was applied to slides and covered with a 22 × 22 coverslip. Sample and probe were denatured at 75°C for 2 minutes, followed by hybridisation at 37°C overnight. Slides were then washed in 2 × SSC (pH 7.0) for 5 minutes shaking, then transferred to 0.4 × SSC (pH 7.0) at 72°C for 2 minutes, followed by another wash in 2 × SSC/0.05% Tween20 for 30 minutes shaking, and a final wash in 1 × PBS for 5 minutes. Detection and signal amplification of biotin‐labelled probes (WCPX and RP11‐195E4) were carried out using Cy3‐conjugated avidin. Slides were counterstained with 4′,6‐diamidino‐2‐phenylindole (DAPI, Vectashield, Vector Laboratories, Peterborough, UK). Single or dual‐colour FISH images were viewed using Leica DM4000 microscope and were captured using the integrated software Leica Application Suite (LAS). M‐FISH was performed using M‐FISH probe kit 24XCyte (Zeiss, Metasystems) according to the manufacturer instructions. Images were acquired with Olympus BX60 microscope for epifluorescence equipped with a JAI CVM4 camera (Leica Biosystems, Wetzlar, Germany). In total, 25 karyotypes were analysed, using the Cytovision software (Leica Biosystems, Wetzlar, Germany). Karyotypes are described according to International System for Human Cytogenetic Nomenclature (ISCN, 2016^36^).

**Table 1:** Details of FISH probes used

Probe Name	Ideogram	Location	Type	Direct Labelling	Manufacturer
XCAP 7 long	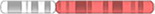	7q	Partial paint	Y	Metasystems
XCAP 7 short	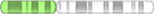	7p	Partial paint	Y	Metasystems
XL7q22/7q36	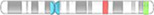	7q22; 7q36	Multicolour single locus	Y	Metasystems
XL MLL plus	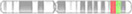	11q23.3	Dual‐colour single locus	Y	Metasystems
XL CDKN2A	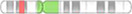	9p21; 9p11‐q11	Dual‐colour single locus	Y	Metasystems
WCP18	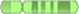	18	Whole paint	Y	Cambio
WCP8	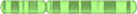	8	Whole paint	Y	Cambio
WCPX	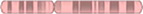	X	Whole paint	N	Cambio
RP11‐195E4	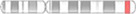	8q24.3	Single colour	N	BACPAC resources center

### 2.3 RT‐PCR and cloning of the *KMT2A*‐*AFF1* fusion

Total RNA was extracted from cell cultures using TRIzol Reagent and converted into cDNA using random hexamers and SuperScript III reverse transcriptase, according to manufacturer's instructions (Invitrogen, Paisley, UK). The *KMT2A‐AFF1* fusion PCR fragment was amplified according to the protocol of van Dongen et al^37^ using the forward primer MLL‐C (5′‐AGGACCGCCAAGAAAAGA‐3′) and the reverse primer AF4‐D (5′‐CGTTCCTTGCTGAGAATTTG‐3′) that anneal to exon 7 on *KMT2A* and exon 7 on *AFF1*, respectively. PCR products were analysed by 2% agarose gel electrophoresis, purified using GeneJET PCR Purification Kit and cloned into pJET2.1 vector (Fisher Scientific, UK). A number of clones containing the insert were selected by colony PCR and then sequenced by GENEWIZ UK Ltd.

## 3 RESULTS

### 3.1 24‐colour karyotyping by M‐FISH

**Figure 1:**
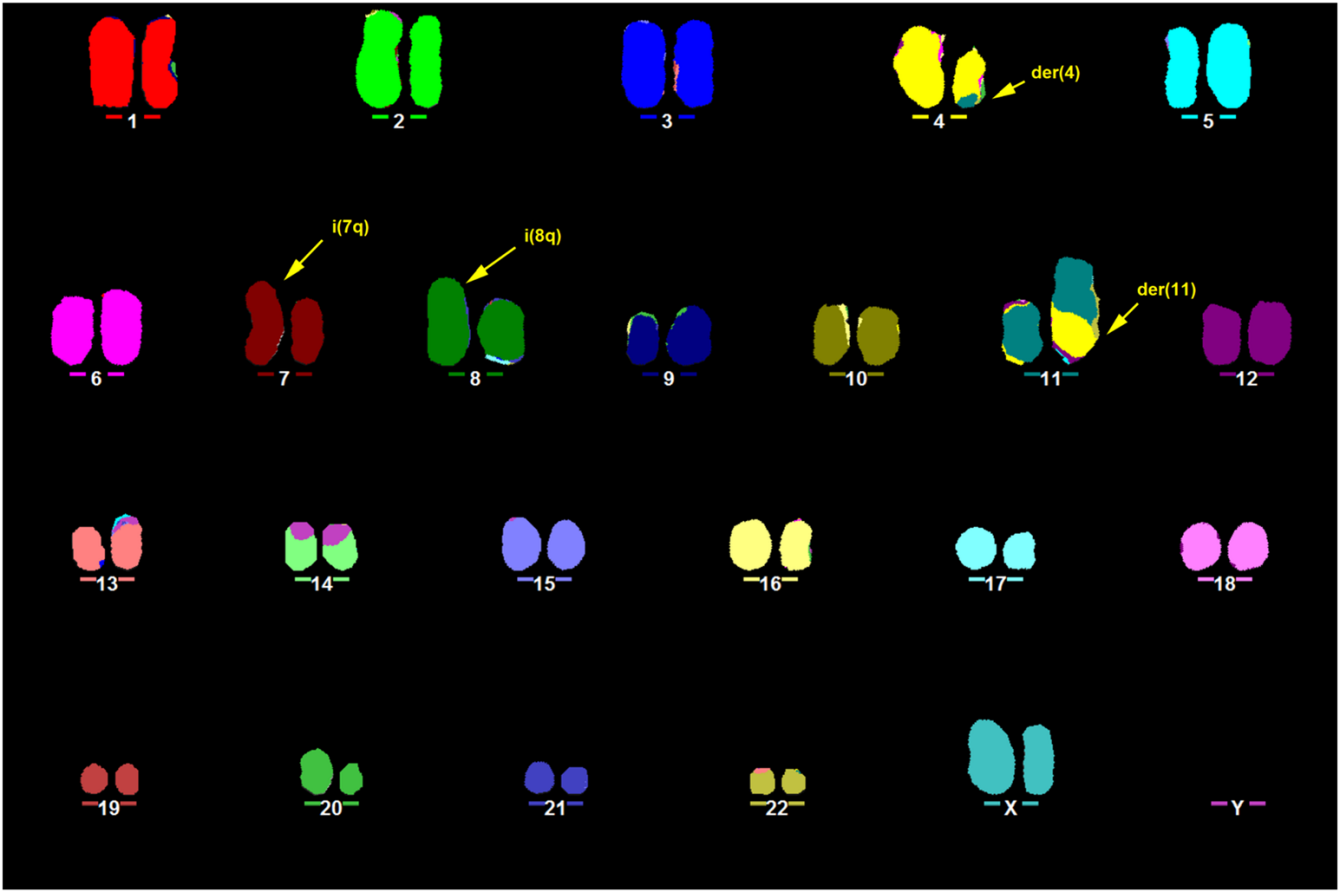
Representative karyotype of RS4;11 obtained by M‐FISH. In this metaphase, the karyotype was determined to be 46,XX,t(4;11)(q21;q23),i(7)(q10),i(8)(q10). Arrows indicate the derivatives der(4) and der(11), i(7q) and i(8q)

All of the metaphases showed the presence of a derivative chromosome 11, carrying chromosome 4 material. In addition, the majority of the cells (76%) also had a derivative chromosome 4 (containing chromosome 11 material in 56% of the cells), and derivatives chromosomes 7 and 8 (Figure 1).

**Figure 2:**
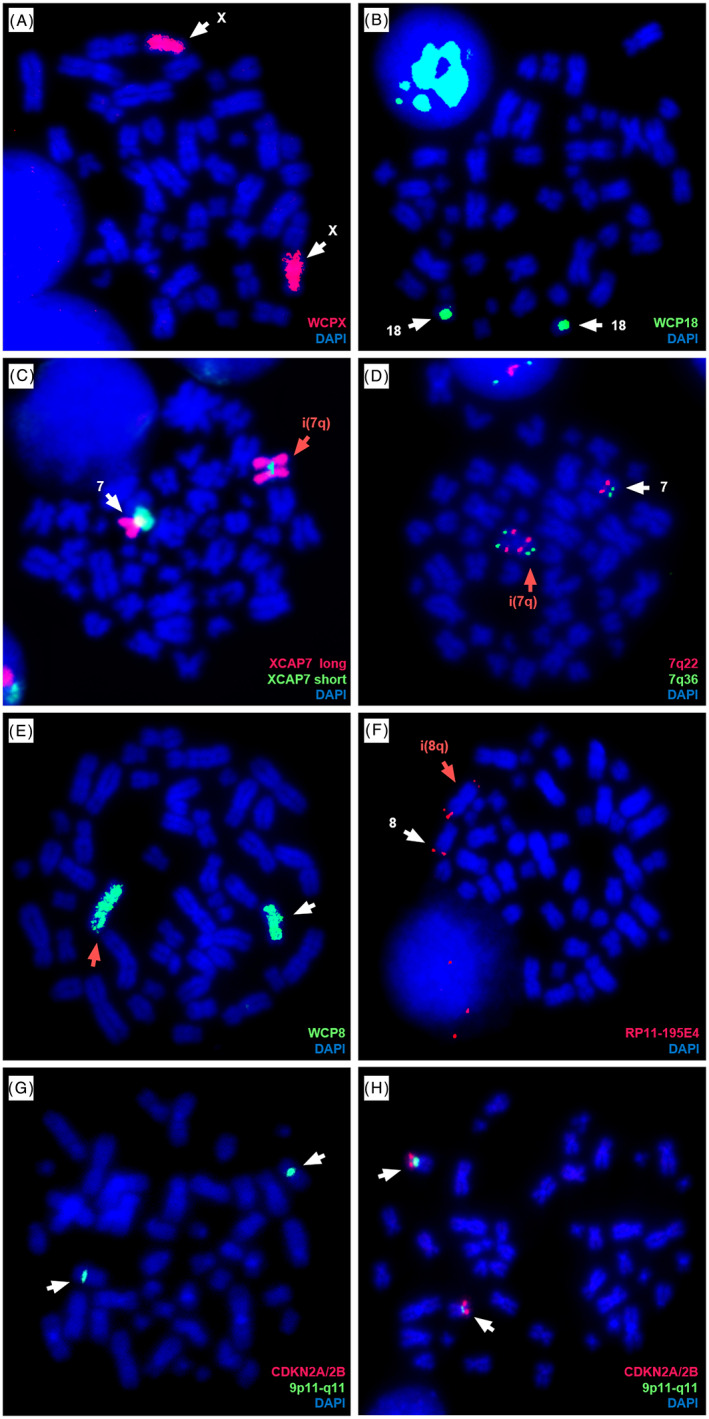
Cytogenetic abnormalities investigated by FISH in the RS4;11 cell line. A‐B, Whole chromosome paints for chromosomes X and 18 did not reveal any numerical abnormalities. C‐D, An isochromosome 7q was detected using an arm‐specific probe (long arm in red and short arm in green), panel C, and a single‐locus probe for 7q22 (red) and 7q36 (green), panel D. E, Whole chromosome paint for chromosome 8 revealed a size difference between chromosomes 8, with one copy appearing metacentric. F, A duplication of the 8q24 region is visible on an isochromosome 8q, detected with the FISH probe RP11‐195E4 specific for 8q24.3. G, Dual‐colour FISH probe CDKN2A/2B XL showed a homozygous deletion of the 9p21 locus by observation of green centromeric signals only. H, A representative metaphase from the Farage cell line with two normal chromosomes 9 with the expected pattern for the CDK24A/2B XL probe

FISH using whole chromosome paints did not reveal any numerical changes or major structural rearrangements in chromosomes 18 and X in 20 metaphases analysed (Figure 2A,B). Analysis of 25 metaphases by M‐FISH reported two cases of +18 but no anomalies for chromosome X. Other numerical changes detected by M‐FISH included +8, −5, −7, −13, −16, and −22.

### 3.2 Confirmation of the major rearrangement t(4;11)(q21;q23) involving *KMT2A*

**Figure 3:**
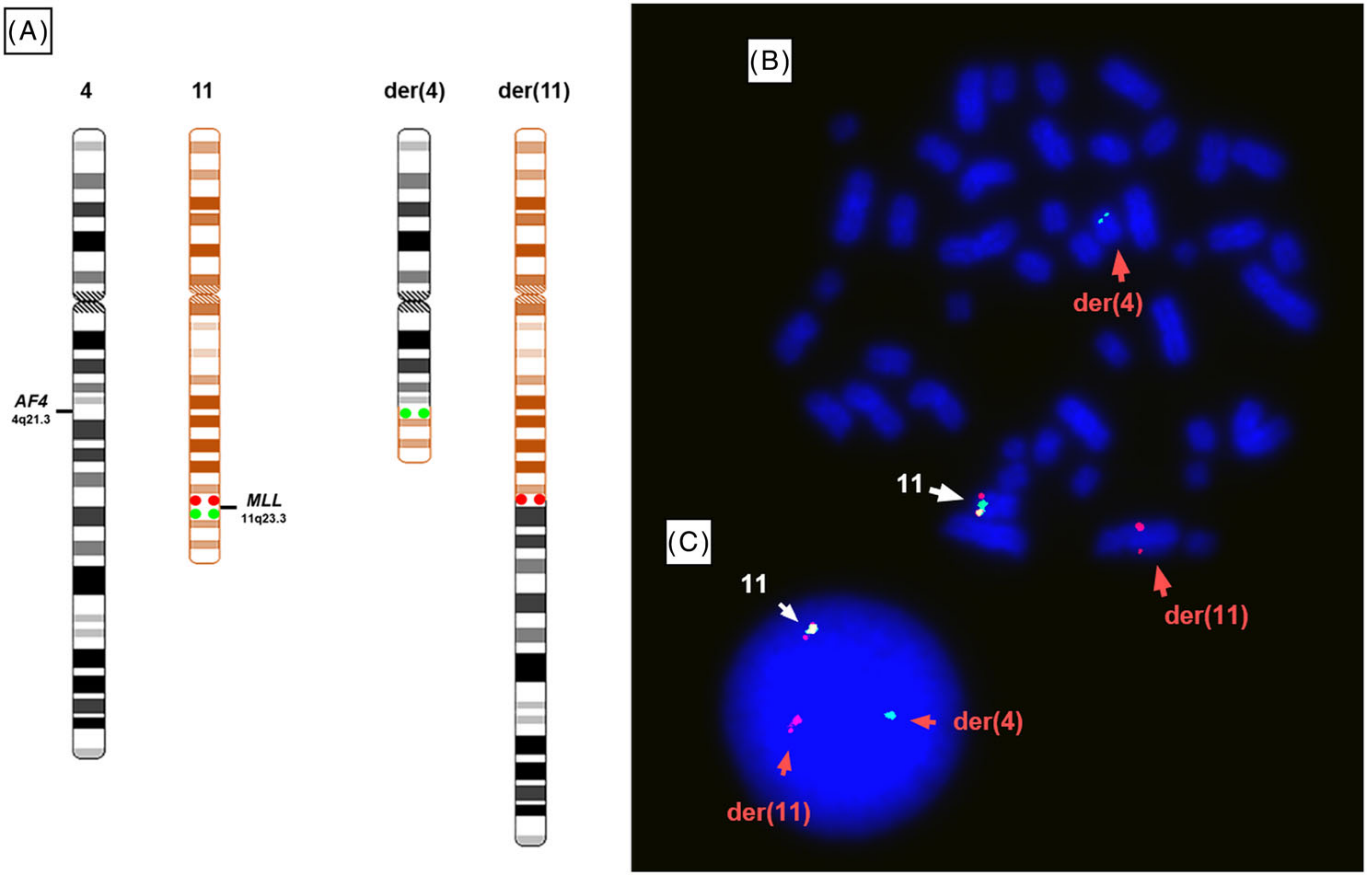
Dual‐colour FISH shows the *KMT2A* rearrangement in RS4;11. FISH using a break‐apart, dual‐colour probe mapping proximal (red) and distal (green) to the *KMT2A* breakpoint region (A) shows the presence of green signals on the der(4), red signals on the der(11), and a yellow fusion signal on the normal chromosome 11 on metaphase chromosomes (B) and in an interphase nucleus (C)

The presence of the t(4;11)(q21;q23) was confirmed by M‐FISH (Figure 1), from which both der(4) and der(11) could be identified. The involvement of the *KMT2A* gene was further confirmed by FISH using a dual‐colour break‐apart probe encompassing the *KMT2A* locus at 11q23.3. One normal *KMT2A* allele could be seen as a yellow fusion signal, whereas disruption of the *KMT2A* region would result in one red and one green signal present on the der(11) and der(4), respectively (Figure 3).

### 3.3 Confirmation of an isochromosome 7q by FISH

The presence of an i(7q) was detected by FISH using arm‐specific chromosome paints for 7q (red) and 7p (green), and single locus probes for bands 7q22 (red) and 7q36 (green). Arm‐specific probes showed the complete coverage of one chromosome 7 in red, corresponding to the long arm (Figure 2C). For single locus probes, this was confirmed by the presence of two additional signals for 7q22 and 7q36 on one chromosome 7 (Figure 2D).

### 3.4 Identification of an isochromosome 8q leading to duplication of 8q24

M‐FISH (Figure 1) and FISH using WCP8 (Figure 2E) revealed two copies of chromosome 8 different in size and centromere position. FISH using a single locus DNA probe for 8q24.3 (RP11‐195E4) was carried out to investigate a possible duplication of this locus. Signals specific for this region were visible on opposite sides of the centromere, suggesting the presence of an isochromosome 8q (Figure 2F).

### 3.5 Homozygous deletion of 9p21 detected by FISH

The dual‐colour probe XL CDKN2A/2B highlighted the presence of both chromosome 9 centromeres. However, lack of both signals for 9p21 revealed a homozygous deletion of that region (Figure 2G,H).

### 3.6 Confirmation of the expression of *KMT2A*‐*AFF1* fusion by RT‐PCR

**Figure 4:**
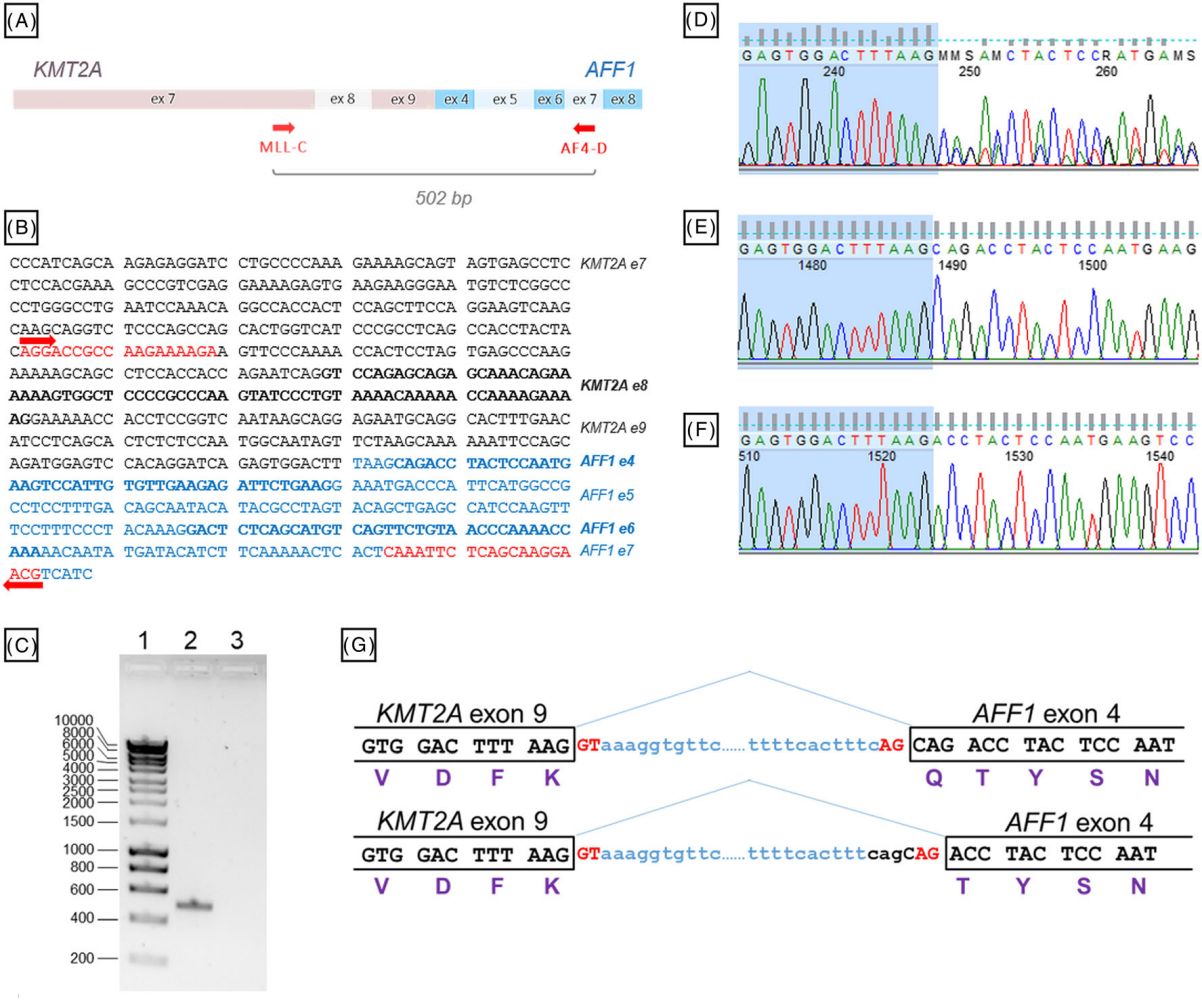
The presence of the *KMT2A‐AFF1* transcript in RS4;11 cells confirmed by RT‐PCR and sanger sequencing. A, Schematic representation of the position of primers flanking the fusion fragment by the forward MLL‐C and reverse AF4‐D. B, The predicted sequence of the RT‐PCR product amplified by the MLL‐C and AF4‐D primers (shown in red text and arrows) was estimated to be 502 bp in size. Accession numbers in Ensembl for the *KMT2A* transcript: ENST00000534358.5; and for *AFF1* transcript: ENST00000307808.10. C, Agarose gel electrophoresis of the amplified fusion product (lane 2), alongside with the nontemplate control (lane 3). Molecular weight markers from the Bioline HyperLadder I are shown in bp (lane 1). D, E, F, Representative sequencing chromatograms of the *KMT2A‐AFF1* junction in the PCR product (D) and in two distinct clones (E, F). The nucleotides of *KMT2A* exon 9 are highlighted in blue. The chromatogram was generated using FinchTV software. G, Schematic representation of the *KMT2A* exon 9‐intron‐*AFF1* exon 4 boundaries demonstrating how the alternative splicing can generate a canonical *KMT2A‐AFF1* transcript, corresponding to the chromatogram in (E) or the transcript containing the deletion of three nucleotides at the beginning of *AFF1* exon 4 that corresponds to the chromatogram in (F)

To confirm that the *KMT2A‐AFF1* fusion mRNA is produced in RS4;11 cells, we amplified the cDNA fragment from exon 7 of *KMT2A* gene to exon 7 of *AFF1* gene, according to the protocol of van Dongen et al^37^ using cDNA generated from total RNA. Agarose gel electrophoresis analysis demonstrates a successful amplification of the cDNA fragment, which migration on the gel is consistent with the in silico calculated size for the amplified *KMT2A‐AFF1* fusion of 502 bp (Figure 4A‐C). Sequencing of the PCR product revealed a mixed sequence starting after the last nucleotide of exon 9 of *KMT2A* gene (Figure 4D), indicating that it is spliced to the different 3′ splice sites. To confirm the presence of alternatively spliced isoforms and estimate the frequency of the alternative splicing event, we cloned the PCR product into the pJET2.1 vector and sequenced a number of clones. Analysis revealed that 8 out of 11 sequenced clones match the sequence of a canonical fusion transcript from the last nucleotide of exon 9 of *KMT2A* gene to the first nucleotide of exon 7 of *AFF1* gene (Figure 4E). Three out of 11 clones showed a deletion of the first three nucleotides (CAG) at the beginning of exon 7 of *AFF1* gene (Figure 4F). The deletion does not cause changes in the reading frame, but results in the loss of a glutamine (Q) residue in the translated sequence, producing two distinct protein isoforms differing in one amino acid (Figure 4G). The genomic sequence at the beginning of exon 7 of the *AFF1* gene has a NAGNAG motif that contains two 3′ splice sites in tandem. The NAGNAG is often subjected to alternative splicing^38^, ^39^; therefore, the detection of noncanonical isoform is not very surprising. Nevertheless, it is important to stress that both transcripts are the results of pre‐mRNA splicing from exon 9 of *KMT2A* to exon 7 of *AFF1*.

## 4 DISCUSSION

The RS4;11 cell line was first established by Stong et al^34^ from the leukaemic cells of a 32‐year‐old female patient with ALL at relapse. Consistent with the original karyotype of the cell line, we identified the characteristic translocation between chromosome 4 and 11, namely t(4;11)(q21;q23), and the isochromosome of the long arm of chromosome 7, namely i(7q). However, i(7q) was not present in the initial karyotype of the patient at diagnosis, but arose as a secondary abnormality in relapse, when RS4;11 was established.^40^, ^41^ In light of our results, the karyotype of RS4;11 is now revised as 47,XX,t(4;11)(q21;q23),i(7)(q10),i(8)(q10),del(9p21)x2/idem,+8/idem,+18.

Additional numerical abnormalities have been previously described in RS4;11, notably trisomy 8 and 18, and monosomy X.^42^, ^43^ In our study, although FISH using whole chromosome paints for chromosomes 8, 18, and X did not confirm these aneuploidies, two separate clones with +8 and +18, respectively, were identified by M‐FISH.

Trisomy of chromosomes 8 and 18 can arise in extended cell cultures and seems to confer a proliferative advantage by an increased gene dosage effect.^44^, ^45^ Trisomy 8 is present in the *KMT2A*‐rearranged cell line MV‐4‐1^46^ and in a number of myeloid leukaemia cell lines such as K‐562,^47^ SKK‐1,^48^ and GDM‐1.^49^ Trisomy 18 is more common in cell lines derived from solid tumours.^44^ In leukaemia patients, trisomy 8 is usually described in therapy‐related leukaemia or as a secondary clonal event,^50^, ^51^ with a possible role in disease progression rather than in primary leukaemogenesis.^52^ Although rare, trisomy 18 in leukaemia is mainly found in conjunction with other abnormalities, such as trisomy 12 or 16 in chronic lymphocytic leukaemia (CLL), and is also regarded as an event of clonal evolution.^53^, ^54^

We report the consistent presence of an i(8q) in RS4;11, which has not been previously described. The formation of the i(8q) results in a duplication of the proto‐oncogene *c‐Myc* mapping at 8q24.2, which has been shown to provide selective growth advantage in vitro.^55^, ^56^, ^57^ In AML and ALL, i(8q) is considered to arise as a secondary clonal abnormality contributing to disease progression and is often found in conjunction with complex karyotypes.^58^, ^59^

We confirmed the homozygous deletion at 9p21, a locus containing *CDKN2A* and *CDKN2B*, coding for the tumour suppressors p16(INK4A)/p14(ARF) and p15(INK4B), respectively. This was previously shown by Southern blotting^60^ and by array‐comparative genomic hybridisation (aCGH).^43^ As part of a large analysis of copy number abnormalities (CNA) in more than 80 B‐ALL cell lines, Tomoyasu et al^61^ confirmed the deletion of the 9p21 region in RS4;11 and also highlighted an overall high frequency of this deletion in the cell lines analysed but a lower frequency in *KMT2A*‐rearranged cell lines. Clinically, homozygous del(9p21) is predominantly found in T‐ALL patients and particularly in paediatric cases, in which the deletion confers poor prognosis.^62^, ^63^ The del(9p21) in conjunction with t(4;11) or other *KMT2A* translocations are seen but at a lower frequency than in other cytogenetic subgroups such as t(1;19) and t(9;22)/*BCR‐ABL*, indicating that the inactivation of *CDKN2A/2B* is not indispensable for the malignant phenotype.^64^

At the molecular level, known breakpoints on *KMT2A* span the region between exon 7 and exon 11, and in *AFF1* the breakpoint locates between exon 8 and exon 4.^9^, ^37^ In RS4;11 the *KMT2A* breakpoint has been shown to occur between exon 8 and 9 and between exon 5 and exon 4 in *AFF1*.^37^, ^65^ We confirmed the in‐frame fusion of the two genes at these breakpoints generating the *KMT2A‐AFF1* transcript. Two distinct isoforms of the fusion are produced differing in a glutamine (Q) residue proximal to the breakpoint. Interestingly, up to eight different transcript variants are known to occur in patients with t(4;11), although their significance has not been elucidated.^66^, ^67^, ^68^, ^69^, ^70^ The NAGNAG motif, identified on exon 7 of the *AFF1* gene, is estimated to be present in 30% of human genes, and it was suggested that it may play a functional role in about 5% of the genes.^71^ Analysis of an EST‐derived alternative splicing database revealed that the NAGNAG motif is indeed subjected to alternative splicing in about 50% cases,^38^ and moreover, the CAGCAG is a consensus sequence.^38^, ^39^ As the NAGNAG often undergoes alternative splicing,^38^, ^39^ the generation of noncanonical isoforms of the *KMT2A‐AFF1* transcripts is unsurprising.

The generation of the reciprocal fusion transcript *AFF1‐KMT2A* from the der(4) was not investigated in our study, but its production has been reported in RS4;11.^72^ The role of the *AFF1*‐*KMT2A* protein in leukaemogenesis is still debated, as *AFF1‐KMT2A* transcripts are only occasionally found in patients due to the fusion not consistently occurring in‐frame.^73^ While some authors have shown that *AFF1*‐*KMT2A* is required to achieve full malignant transformation,^74^, ^75^ others did not find such association.^72^, ^76^

Although at least 16 cell lines with the t(4;11) have been described, only few have been appropriately authenticated (summarised in Table 2).^32^, ^33^ A similar cell line to RS4;11 is MV‐4‐11, harbouring a t(4;11)(q21;q23) together with an additional copy of chromosome 8 and chromosome 19.^84^ MV‐4‐11 is morphologically macrophagocytic and was established from a 10‐year‐old male patient with biphenotypic myelomonocytic leukaemia.^46^ While RS4;11 serves as a model for pre‐B lymphoblastic leukaemia, MV‐4‐11 caters well for studies on myeloid and myelomonocytic cells.^33^ The SEM cell line also carries a t(4;11), in conjunction with a del(7)(p14) and del(13)(q12), and was established from a 5‐year‐old female ALL patient.^82^ AN4;11 was derived from a 8‐month‐old diagnosed with T‐ALL with t(4;11) as the sole abnormality reported.^83^ Finally, the B‐1 cell line was derived from a relapsed ALL 14‐year‐old male with a more complex karyotype compared with the other t(4;11) cell lines. B‐1 harbours a t(4;11) without a der(4), as well as t(1;8)(p36;q13), t(1;10)(q11;p15), monosomies 4 and 9, and trisomy 6.^80^ All cell lines generate the *KMT2A‐AFF1* and the reciprocal *AFF1‐KMT2A* fusions,^85^ with the exception of the B‐1 cell line, which only produce the *KMT2A‐AFF1* from the der(11).^79^, ^80^ In terms of immunophenotypic profiling, they are all defined as biphenotypic based on the co‐expression of lymphatic and myeloid antigenic markers CD10, CD19, CD13, CD34, and CD33.^86^

**Table 2:** Features of cell lines with t(4;11)

Cell Line	Diagnosis	Age/Gender	Cell Type/Morphology	Original Karyotype	*KMT2A‐AFF1*	*AFF1‐KMT2A*	Other Molecular Features	Ref.
RS4;11	B‐ALL at relapse	32y/F	Lymphoblast	46,XX,t(4;11)(q21;q23),i(7q)	Y	Y	WT *TP53* 77	34
MV‐4‐11	Biphenotypic B‐myelomonocytic leukaemia (AML)	10y/M	Macrophage/lymphoblast	48,XY,t(4;11)(q21;q23),+8,+19	Y	Y	*FLT3* ITD^78^; WT *TP53* 77	46
B‐1	ALL at relapse	14y/M	Lymphoblast	45,XY,t(4;11)(q21;q23),t(1;8)(p36;q13),t(1;10)(q11;p15),−4,+6,−9	Y	N – der(4) absent^79^		80
SEM	B‐ALL at relapse	5y/F	Lymphoblast	45,XX, t(4;11)(q21;q23),del (7)(p15),−13	Y	Y	*FLT3* iAMP^81^	82
AN4;11	T‐ALL	8 m/M	Lymphoblast	46,XY,t(4;11)(q21;q23)	Y	Y		83

Abbreviations: iAMP, intrachromosomal amplification; ITD, internal tandem duplication.

The MV‐4‐11, SEM, AN4;11, and B‐1 cell lines are particularly suitable for the study of childhood *KMT2A* leukaemia, as they were established from paediatric patients, whereas RS4;11 can be considered a model to investigate adult relapsed forms. Studies on these cell lines have also helped decipher key players in leukaemogenesis. For instance, the absence of the der(4) in the B‐1 cell line, which is however present in MV‐4‐11, RS4;11, AN4;11, and SEM, suggests that it is the der(11) to detain a pivotal role in the promotion and maintenance of the malignancy.^79^, ^80^ Differences in other molecular features, such as mutations of known cancer‐related genes *TP53* and *FLT3*, can prove useful in investigating the role of point mutations in leukaemogenesis^77^, ^78^, ^81^ (Table 2).

We conclude that the RS4;11 cell line is a suitable in vitro model for the study of leukaemia harbouring t(4;11), owing to the presence and retention of its original cytogenetic abnormalities. At the molecular level, the production of the *KMT2A‐AFF1* transcript is useful for proteomic and pharmacological studies, and might help to shed some light on the significance of transcript variants and alternative splicing. As with other cell lines, the karyotype is subjected to evolution over extended culturing, by the accumulation of secondary mutations and rearrangements. Monitoring cell lines over time would provide further information on clonal evolution and proliferative advantage of certain subclones, such as the proportion of different clones within the population, how they vary over time, and which abnormalities provide the strongest growth advantage. While these changes may represent an obstacle in reproducibility and reliability of experiments, investigating these changes could be used to understand tumour evolution in patients.^87^, ^88^

## CONFLICT OF INTEREST

The authors have no conflict of interest.

## AUTHORS' CONTRIBUTION

All authors had full access to the data in the study and take responsibility for the integrity of the data and the accuracy of the data analysis. *Conceptualisation*, S.T.; *Methodology*, S.T., E.M.M.; *Investigation*, D.R., O.B., D.M., E.M.M., *Formal analysis*, D.R., O.B., D.M., E.M.M., S.T., *Resources*, S.T., E.M.M., C.M.G.; *Writing—original draft*, D.R., S.T., E.M.M., D.M.; *Writing—Review and Editing*, D.R., S.T., E.M.M., D.M.; *Visualisation*, D.R., S.T., E.M.M., D.M.; *Supervision* S.T., E.M.M., C.M.G.; *Project Administration*, S.T.

## Data Availability

The data that support the findings of this study are openly available in https://brunel.figshare.com at https://doi.org/10.17633/rd.brunel.8016191.
